# Type IV Hypersensitivity Reaction after Cyanoacrylate Venous Closure

**DOI:** 10.1016/j.avsg.2023.06.002

**Published:** 2023-06-08

**Authors:** Leigh Ann A. O’Banion, Michael Y. Shao, Amna Ali, Mariya Kochubey, Yueqi Yan, Joshua Fallentine, Jae Hak Oh, Harik R. Patel, Nishant Agrawal, Emely Carmona, Eric S. Hager, Misaki M. Kiguchi

**Affiliations:** 1 Division of Vascular Surgery, Department of Surgery, University of California San Francisco-Fresno, Fresno, CA.; 2 Division of Vascular Surgery, Northshore University Health System, Chicago, IL.; 3 University of California Merced, Merced, CA.; 4 Georgetown University School of Medicine, Washington, DC.; 5 St. George’s University of London, London, UK.; 6 Division of Vascular Surgery, Department of Surgery, University of Pittsburgh Medical Center, Pittsburgh, PA.; 7 Department of Vascular Surgery, MedStar Washington Hospital Center, Washington, DC.

## Abstract

**Background:**

Nonthermal endovenous closure techniques are routinely utilized to treat superficial axial venous reflux. Cyanoacrylate closure is a safe and effective modality implemented for truncal closure. However, an adverse reaction of type IV hypersensitivity (T4H), unique to cyanoacrylate, is a known risk. This study aims to evaluate the real-world incidence of T4H and examine risk factors that may predispose its development.

**Methods:**

A retrospective review between 2012– and 2022 was performed at four tertiary US institutions to examine patients who underwent cyanoacrylate vein closure of their saphenous veins. Patient demographics, comorbidities, CEAP (Clinical [C], Etiological [E], Anatomical [A], and Pathophysiological [P]) classification, and periprocedural outcomes were included. The primary endpoint was development of T4H post procedure. Logistic regression analysis for risk factors predictive of T4H was performed. Variables with a *P*-value of <0.05 were deemed significant.

**Results:**

595 patients underwent 881 cyanoacrylate venous closures. Mean age was 66.2 ± 14.9, and 66% of patients were female. There were 92 (10.4%) T4H events in 79 (13%) patients. Oral steroids were administered to 23% for persistent and/or severe symptoms. There were no systemic allergic reactions to cyanoacrylate. Multivariate analysis revealed younger age (*P* = 0.015), active smoking status (*P* = 0.033), and CEAP 3 (*P* < 0.001) and 4 (*P* = 0.005) classifications as independent risk factors associated with development of T4H.

**Conclusions:**

This real-world multicenter study shows the overall incidence of T4H to be 10%. CEAP 3 and 4 patients of younger age and smokers predicted a higher risk of T4H to cyanoacrylate.

## INTRODUCTION

With the well-established significant healthcare burden of over 2 billion US dollars chronic venous insufficiency imparts, technologies to improve the treatment of superficial venous reflux are ever-evolving to be more effective and efficient.^1,2^ While historically high ligation and stripping were standard of care techniques, minimally invasive approaches largely performed in the outpatient care setting now predominate.^3^ Thermal ablation of superficial veins has been the predominant treatment of choice for endovenous ablation of the saphenous system for several decades now, but it is limited in its treatment length due to the risk of thermal nerve injury.^4^ In efforts to resolve this limitation and provide broader techniques to completely treat the entire length of refluxing saphenous vein, newer nonthermal modalities have been developed.^5,6^

Cyanoacrylate glue closure (CAC) is one such device that utilizes a delivery gun to deposit small aliquots of glue along the length of the saphenous vein through a catheter-based system. The cyanoacrylate glue is considered an implantable device which polymerizes, causing occlusion and thrombosis of the saphenous vein. This technology has been approved by the Food and Drug Administration (FDA) since 2017, and data supports both its safety and efficacy with rates of endovenous glue induced thrombosis (EGIT) as low as 1.3% in large multicentered studies and closure rates approaching 98% in the literature.^7–9^ However, a unique reaction specific to the cyanoacrylate glue has been observed. Type IV hypersensitivity reaction (T4H) after CAC was first described by Gibson et al. in a single institutional analysis of 313 procedures from the WAVES and VeClose trials.^10^ It was defined as “red, itchy dermal reaction that is sometimes painless, but sometimes associated with discomfort and/or localized swelling.” The authors identified a 6% incidence overall with 28% of these cases requiring oral steroids. The authors, however, did not identify any significant risk factors for its development. Since its publication, the data on T4H events has been sparse, limited to single institutional studies and case reports.^11^ We hypothesize that the real-world incidence of T4H may be higher than reported in these smaller studies and aim to identify significant risk factors that may aid in improved patient selection and education for treating physicians.

## METHODS

A multi-institutional retrospective review of all patients who underwent cyanoacrylate closure of their saphenous veins from 2017–2022 at four tertiary care US institutions were examined. This study was approved by the institutional review board at Community Medical Centers, UCSF-Fresno and data use agreements were employed where necessary. Patient demographics, comorbidities, CEAP (Clinical [C], Etiological [E], Anatomical [A], and Pathophysiological [P]) classification, venous physiologic duplex data, and periprocedural outcomes were extracted from the individual institutional electronic health records.

All venous reflux patients at their respective institutions were evaluated with a complete history and physical exam, placed in compression therapy, and underwent a complete venous duplex interrogation performed by a certified vascular sonographer. Criteria for pathologic superficial venous insufficiency are standardized according to the Society for Vascular Surgery clinical practice guidelines and defined reflux >0.5 seconds for all axial veins with a diameter exceeding 3.5 mm.^3^ If patients were deemed candidates for venous intervention, various thermal and nonthermal ablative techniques available at the respective institution were discussed. Ultimately, the procedure choice was made by a shared patient and provider decision as well as insurance authorization. Patients with known allergies to cyanoacrylate or acrylic derivatives were not offered cyanoacrylate ablation.

Cyanoacrylate ablation was carried out according to instructions for use as described by the manufacturer via antegrade single puncture access on all patients. Patients were not routinely premedicated with steroids, antihistamines, or prophylactic anticoagulation prior to ablation. All patients followed up with postprocedural duplex imaging within 14 days of their procedure. The development of a T4H reaction was defined as a “red, itchy dermal reaction that is sometimes painless but sometimes associated with discomfort and/or localized swelling,” consistent with prior studies. Upon diagnosis, patients were instructed to take nonsteroidal anti-inflammatories as well as diphenhydramine orally as needed for symptoms. Prescription for oral steroid administration (standard methylprednisolone 5-day taper) was offered when severe and/or persistent symptoms were based on physician discretion.

The primary endpoint was development of T4H post procedure, documented in the medical record. A secondary endpoint was needed for oral steroid administration. Descriptive statistics compared the sample characteristics of patients with developed T4H to those without. Chi-square tests or Fisher’s exact tests were performed for categorical variables, and *t*-test was used for continuous variables. Logistic regression examined factors associated with T4H. A robust standard error was used to adjust for clustering within patients (i.e. patients with multiple cyanoacrylate ablations). Bivariate and fully adjusted models included all demographic and clinical factors to identify those independently associated with the risk of T4H after accounting for potentially confounding variables. Statistical significance was defined as a *P* value < 0.05.

## RESULTS

A total of 595 patients underwent 881 cyanoacrylate saphenous closures. The mean age was 66.2 ± 14.9 and 397 (66.7%) were female. The majority of the patient population was either White (51%) or Hispanic (28.2%) and predominantly obese (average body mass index (BMI) 31 kg/m^2^ ± 7.6). Baseline demographics, medication allergies, thrombotic history, and comorbidities are outlined in Table I. Out of a total of 881 procedures, there were 92 (10%) documented T4H events in 79 (13%) patients. There were no documented systemic allergic reactions (anaphylaxis, hypotension, hives, etc.) to the cyanoacrylate adhesive. Of the 79 patients with T4H, 18 (22.8%) were prescribed steroids for persistent and/or severe symptoms. All patients had complete resolution of the reaction, and no patient required vein excision.

In a univariate analysis examining risk factors for development of T4H, only younger age (*P* = 0.001), advanced CEAP classification (*P* < 0.001), and vein treated patients (*P* = 0.016) were significantly associated with the development of T4H. Table I. On further investigation, in a multivariate logistic regression, younger age (OR 0.98, 95% CI 0.96, 1.00), active smoking status (OR 2.4, 95% CI 1.07, 5.34), and CEAP 3 (OR 4.2, 95% CI 1.95, 8.86) and CEAP 4 (OR 3.6, 95% CI 1.48, 8.74) classification were independently associated with the development of T4H. Table II.

## DISCUSSION

The recent adoption of cyanoacrylate closure of refluxing saphenous veins in 2017 has perceived benefits of safely and effectively treating the above and below the knee saphenous vein in its entirety without nerve injury, as compared to thermal techniques.^8^ In addition, this nonthermal technique avoids all tumescent anesthesia, use of postprocedural compression stockings, and the ability to return to work faster than its thermal counterparts.^12^ Venous-specific quality of life measures, also, are improved across diverse patient populations.^13,14^ These advantages, however, require balance against any unique risks and in this case, T4H.

Despite the safety and efficacy demonstrated by the VeClose trial, T4H was described in the WAVES trial.^7,15,16^ Further frequency and severity of T4H after cyanoacrylate closure were explored by Gibson et al. Out of 379 limbs treated in 286 patients, T4H occurred in 18 patients (5.8% treatments; 6/3% patients).^10^ Contrary to our study, advanced CEAP classifications (4–6) patients trended toward decreased T4H. Interestingly, 27 patients with T4H had repeat intervention on a second limb, without additional T4H. Similar to this multi-institutional real-word study, the majority of reactions were self-limited, resolving with conservative measures or oral steroids in a small subset.

In a study of predominantly Asian ethnicity, the incidence of T4H occurs more frequently. This retrospective review of 126 saphenous veins treated with cyanoacrylate by Sermsathanasawadi et al. showed an incidence of 15.8% in 15% of limbs. Although all symptoms were mild and treated with nonsteroidal anti-inflammatory drugs, with resolution within a week, significant risk factors included suprafascial saphenous vein of <1 cm and saphenous veins >8 mm.^17^ Park et al. also described a “phlebitis-like” reaction in 25.4% of veins treated, which is higher than previously reported T4H rates in the US and Europe.^11^ In Singapore, Tang et al. reported T4H in 18% of treated patients.^14^ The higher incidence could be attributed to variation in reaction definition but could also be attributed to some ethnic differences in inflammatory response. All “phlebitis-like” reactions were treated with nonsteroidal anti-inflammatory agents without the need for excision.

It is hypothesized that this “reaction” to cyanoacrylate is due to a T4H, mediated by T-cells.^11,18,19^ The patients within this study who had T4H were routinely managed with nonsteroidal anti-inflammatory for a short duration, with only a small portion requiring a short steroid taper for complete resolution of symptoms. Patch testing prior to cyanoacrylate closure may suggest patients who are susceptible to T4H. However, this is off-label. As mentioned prior, many of the patients who underwent repeat intervention despite having an initial T4H did not have a subsequent reaction.

Although severity is widely variable, a significant portion (10%) of patients undergoing cyanoacrylate closure experienced some degree of T4H. This reaction, unique to cyanoacrylate adhesive, needs to be a recognized as a potential adverse event, identified swiftly and treated early, if necessary. Vein excision is reported to be rare as early recognition and treatment with nonsteroidal anti-inflammatories and/or steroids appears to quickly resolve this reaction.^20,21^ Patients in this study were not routinely given nonsteroidal anti-inflammatories periprocedurally, but this could be considered into practice protocol to decrease T4H rates in patients undergoing cyanoacrylate closure, if no medical contraindications. In addition, the authors hypothesize that the avoidance of cyanoacrylate in patients with chronic autoimmune conditions, including asthma, arthritis, lupus, etc. may decrease the incidence, although the power of this study was not such to detect a statistical significance. These individuals may incur higher rates of T4H, as they are often more susceptible to immunological insult, and further investigation on the pathophysiology behind T4H should explore a possible connection. Smoking, known to cause inflammatory response within the vasculature, proved to be a risk factor in this study as a predictor of T4H, additionally supporting a possible connection between predisposition to T4H within patients with already existing proinflammatory states.

Furthermore, the risk factors for T4H found in this study, including smoking, younger age, and advanced CEAP classification, may also modify practice patterns by employing careful patient selection and shared decision-making through patient education on the potential adverse side effects of cyanoacrylate. However, due to other studies with controversial findings of opposite CEAP relationships, further higher-powered studies may be necessary to delineate the relationship between CEAP classification and T4H risk. Additionally, there is sparse data on the epidemiology of delayed T4H reactions in the general population, and thus it is difficult to make generalized conclusions about why our younger patient population reported a higher incidence of T4H reaction.^22^ Regardless, the T4H incidence in all of these studies were mild and self-limiting and were treated medically with over-the-counter nonsteroidals in a large majority, supporting the safety of cyanoacrylate closure.

Potential limitations to this study include the retrospective nature and its inherent biases compared to blinded randomized studies. Furthermore, while the definition of T4H was standard across institutions, variation in patient reporting and lack of patient health literacy, transient nature of symptoms, and inconsistent follow-up may all attribute to underreporting of T4H incidence. Further studies need to explore patient and procedural risk factors that may improve patient selection to mitigate T4H and the associated improvement in patient reported outcomes elucidated from improved patient education and selection.

## CONCLUSION

This multicenter study demonstrates the overall incidence of T4H in the US population to be 10%. This promising technology with the ability to eliminate the entire length of venous reflux without nerve injury should be balanced with possible T4H that occurs uniquely with cyanoacrylate in certain populations. CEAP 3 and 4 patients of younger age and smokers predicted higher risk of T4H to cyanoacrylate. All reactions were self-limited, and a minority of patients required oral steroid administration for resolution, suggesting an overall benign nature of this reaction, which may be, mitigated in select high-risk patients though careful selection and education.

## Figures and Tables

**Table I.** Demographics and descriptive analysis of risk factors for type IV hypersensitivity (T4H)

By patient	No T4H reaction (*N* = 516 patients)	T4H reaction (*N* = 79 patients)	Total (*N* = 595 patients)	*P* Value
Age	66 ± 14.9	61 ± 14.3	66 ± 14.9	0.001
BMI	31.0 ± 7.7	30.9 ± 6.8	31.0 ± 7.6	0.836
Female sex	339 (65.7%)	58 (73.4%)	397 (66.7%)	0.175
Medication allergies (Yes)	192 (37.2%)	34 (43%)	226 (38%)	0.320
Number of allergies	0.8 ± 1.7	0.7 ± 1.1	0.8 ± 1.6	0.528
Autoimmune disorder	4 (0.8%)	1 (1.3%)	5 (0.8%)	0.511
History of SVT	20 (3.9%)	2 (3.5%)	22 (3.7%)	0.755
History of DVT	48 (9.3%)	4 (5.1%)	52 (8.7%)	0.214
Hypercoaguable disorder	8 (1.6%)	3 (3.8%)	11 (1.9%)	0.167
Type II diabetes	126 (24.4%)	20 (25.3%)	146 (24.5%)	0.863
Active smoker	41 (8.0%)	11 (13.9%)	52 (8.7%)	0.808
By procedure	No T4H Reaction (*N* = 782 procedures)	T4H Reaction (*N* = 92 procedures)	Total (*N* = 881 procedures)	*P* value
CEAP classification				<0.001
2	215 (27.3%)	12 (13%)	227 (25.8%)	
3	227 (28.8%)	49 (53.3%)	275 (31.3%)	
4	163 (20.7%)	26 (28.3%)	189 (21.5%)	
5	19 (2.4%)	1 (1.1%)	20 (2.3%)	
6	165 (20.9%)	4 (4.4%)	169 (19.2%)	
Maximum vein diameter (mm)	6.5 ± 19.9	6.9 ± 2.8	6.5 ± 18.8	0.833
Vein treated				0.016
AASV	42 (5.3%)	5 (5.4%)	47 (5.3%)	
GSV	579 (73.4%)	79 (85.9%)	658 (74.7%)	
SSV	168 (21.3%)	8 (8.7%)	176 (20.0%)	

SVT, superficial thrombophlebitis; DVT, deep venous thrombosis; AASV, anterior accessory saphenous vein; GSV, great saphenous vein; SSV, small saphenous vein.

**Table II.** Results of logistic regression models in predicting T4H

Variable	Bivariate models		Ajusted models	
	OR [95% CI]	*P*-value	[95% CI]	*P*-value
Age	0.98 [0.96, 0.99]	0.001	0.98 [0.96, 1.00]	0.015
Female sex	1.34 [0.76, 2.35]	0.309	1.31 [0.66, 2.59]	0.444
BMI	1.00 [0.97, 1.03]	0.873	0.99 [0.95, 1.03]	0.595
Number of allergies	0.91 [0.78, 1.07]	0.272	0.91 [0.79, 1.05]	0.201
Autoimmune disorder	1.43 [0.15, 13.36]	0.752	2.42 [0.1, 56.71]	0.584
History of SVT	0.63 [0.14, 2.78]	0.539	0.61 [0.11, 3.38]	0.569
History of DVT	0.45 [0.16, 1.28]	0.134	0.39 [0.14, 1.12]	0.081
Hypercoagulable disorder	2.38 [0.68, 8.41]	0.177	3.85 [0.9, 16.43]	0.069
Type II diabetes	0.96 [0.55, 1.66]	0.881	1.37 [0.69, 2.73]	0.367
Active smoker	2.56 [1.19, 5.50]	0.016	2.39 [1.07, 5.34]	0.033
Vein treated				
AASV	Ref		Ref	
GSV	1.15 [0.47, 2.77]	0.762	1.40 [0.59, 3.32]	0.449
SSV	0.40 [0.13, 1.28]	0.122	0.70 [0.22, 2.26]	0.555
CEAP				
2	Ref		Ref	
3	3.87 [1.86, 8.04]	<0.001	4.16 [1.95, 8.86]	<0.001
4	2.86 [1.32, 6.17]	0.007	3.6 [1.48, 8.74]	0.005
5	0.94 [0.11, 7.92]	0.957	1.66 [0.17, 15.84]	0.658
6	0.43 [0.13, 1.41]	0.164	0.64 [0.17, 2.37]	0.499
Maximum vein diameter (mm)	1.00 [0.99, 1.01]	0.654	1.00 [0.99, 1.00]	0.676

SVT, superficial thrombophlebitis; DVT, deep venous thrombosis; AASV, anterior accessory saphenous vein; GSV, great saphenous vein; SSV, small saphenous vein.
